# On the Diseases of Onondaga County

**Published:** 1849-05

**Authors:** 


*On the diseases of Onondaga County.* By D. T. Jones, M. D., of Baldwinsville, Onondaga County.

Gentlemen:

In obedience to a resolution of this Society, I would submit a few remarks on the most prevolent diseases of the county of Onondaga.

This county is situated in the central part of the state, its southern half is elevated and hilly, lying on the Onondaga group of limestone. The northern half of the county is very level, being north, and below, the outcropping of the limestone group, and scarcely broken by an elevation of any amount.

In consequence of the distinctive feature, few swamps and morasses are found in the southern section of the county, the streams are rapid, with high banks, while in the north, they are sluggish and muddy, with low banks. The Seneca river, a large, deep and sluggish stream, enters the county from the west, and after a slow winding course unites with Oneida river, the outlet of Oneida lake, the two forming the Oswego river, and, at the same time, taking a more rapid course until it falls into Lake Ontario. These rivers have been noted since the first settlement of the county, for the amount of intermittent and remittent fevers found along their border.

This great distinction of surface, has from the first widely separated the character of the diseases prevailing in the northern and southern sections of the county. In the town of Pompey, situated in the south-eastern part of the county, typhus or typhoid has long been the prevailing type of fevers; while physicians to the north, rarely if ever saw the disease, or if so it was the product of importation.

I reside in the village of Baldwinsville, on the north bank of Seneca river, and for the last twenty years have prosecuted my profession there and in its vicinity. As I intend to confine my remarks principally to facts falling under my own observation, they will generally be applied to the low alluvial portion of the county with which I am partially acquainted. In this part of the county, intermittents and remittents, have been the prevailing type of fever until within a few years. Few persons escaped them for more than a year or two, and it would be difficult now to find an old resident who has not had one or the other, more commonly both. Remittents were formerly often fatal, now this is seldom the case. The treatment then, was antimony in emetic, and continued in febrifuge doses, catharthics of calomel and jalap, drastic billious pills, Dover powders, blisters, and a torturing abstinence from cooling drinks.

Now from ten to fifteen grains of quinine, repeated once or twice at intervals of twenty-four hours, given as soon after the cessation of paroxysm of fever as may be, a full anodyne at night, with plenty of cool drinks, a cathartic of calomel as soon after the interruption of the paroxysm as convenient and proper, followed with an occasional pill of blue mass, or blue mass and aloes, completes the cure of either an intermittent or remittent fever. Judging from my own, as well as the experience of others, I am of opinion that quinine approaches as near a specific in the cure of a remittent as of an intermittent fever, and is of infinitely more importance in treating the former than the latter.

This practice has now been continued so long and with such uniform success, that the common people as often speak of breaking up a "bilious remittent" as of the ague.

No state of the tongue, the skin or the bowels, forbid its use, and the more violent the paroxysm the more certain the remedy. The more common errors which are fallen into in this treatment are a too small, and too often repeated use of quinine. In small and often repeated doses this agent harasses and irritates the stomach, an effect sure to be followed by debility, as is often seen in the administration of any of the bitter tonics, for a long time in succession. Another error consists in discharging your patient as cured as soon as the paroxysms of fever have left him. The animal economy has received a violent shock both in the inception and cure of the disease; the effects of both these are to be removed; the secretions are changed and nature must be helped to take on the normal healthy action in all the functions of the body. Until then he errs who pronounces his patient cured.

In the falls of '39 and '40 a fever prevailed which was new to us, and received different names, in various localities where it prevailed, applied as caprice or whim might indicate, to some one of its more prominent symptoms, as gastric, red tongue, black tongue, bilious-typhoid, typhus, and typhoid fever. The symptoms, as this extended nomenclature would indicate, were varied.

We commenced treating it as a bilious remittent fever, but soon found it was governed by other laws. Calomel, antimony, sudorifics and tonics, did no good, but often, very often, much harm. A red, dry and perfectly smooth tongue, a disordered bowel, a dry skin with continued fever would gradually come on, so that at the end of a week we had in truth a typhus fever. However, the disease was fatal but in a very few instances, although it prevailed to an alarming extent.

Physicians soon took the hint, and abandoned the use of heroic remedies, and put their patients upon treatment which might be called expectant, from the commencement, and the result was, that nearly all recovered, but after a long protracted run of from twenty to sixty days.

Since that time up to the last fall, almost all our cases of fever were typhus or typhoid, and in most cases were easily traced to an infectious origin. The disease has been gradually loosening all abnormal symptoms until at present a good synopsis can be found in Cullen's first lines, under the head of Typhus Mitior.

During the early part of the past season we had more of the distinct bilious fever, which has readily given way to the tonic, anodyne and alterative treatmeut. The most prevalent disease throughout the county, in both the summers of '47 and '48, has been dysentery. It has been more obstinate and protracted than usual, but has generally yielded to an anodyne and laxative treatment.

The use of calomel or what is generally termed "the alterative treatment," was not thought to be beneficial but often highly injurious.

My friend Dr. Taylor writes me that he used nitrate of silver in several protracted cases with manifest advantage. The city of Syracuse suffered most, but generally among children. Taking the whole amount of cases I think the disease was not unusually fatal.

Idiopathic jaundice has prevailed the last fall to such an extent as to put on the appearance of an epidemic. It yielded to an alterative and saline treatment in about two weeks, but left the digestion in a weak, irregular and unhealthy condition. Has this epidemic derangement of the digestive organs at this time any thing to do with the state of the atmosphere preceding the appearance of the Asiatic Cholera?

Scarlatina has prevailed in our county for the last eight or ten years, but generally in two sections at the same time. It has been seen in all of its grades, from the most mild to the most malignant, where the symptoms of illness was begining to die.

Typhus fever exists in many parts of our county at this time, and in the village where I reside. It is mild in its form. Commencing with pain in the head and neck; soreness of the muscles of the eyes, neck and extremities; pulse and heat nearly natural; tongue with a heavy white coat of fur; bowels costive at first but soon becoming loose, with a tendency to fullness; deliriums at night; dullness of intellect; slowness and hesitancy of speech; the manner of which is so peculiar that a person acquainted with the disease would detect its existence by the ear alone. It may be fancy but to me the countenance seems to have a peculiar, slightly bloated, and leaden hue look, which holds through the whole course of the disease, be the event what it may. The face appearing nearly as full as in health although the extremities are wasted to a skeleton, the dark color only leaving with confirmed convalescence.

From my own observation and those of my friends who have seen much of the disease we are inclined to thing the disease idiopathic typhus fever, and no new disease, nor indeed a new phasis of typhus fever, but the plain typhus mitior of Cullen's nosological arrangement, a work which has not yet been equalled in its symptomatology. Of disease generally, our county offers nothing peculiar. Of its treatment, we can boast of as great a variety, as any county of equal extent, from the most energetic discipline of Armstrong down — down to the infinitessimal nothingness of Hahnemann. The doctrines of Broussais I think have greatly modified the treatment of disease throughout the civilized world, and the medical practitioner early in life, before

"His lyart haffets wearin thin and bare,"

adopts the cautious practice of age, treating disease on the expectant, rather than the curative plan of Reush and Armstrong. That the treatment of most diseases is much improved, is certain. That we are often too tentative and cautious, I think fully as certain. That our fevers are now mostly typhus and require less depletion than the bilious remittent, no one will for a moment dispute, but that the pain in the head and back, the intense burning fever of a bilious attack, are now as ever, best combatted by an early bleeding, followed by other active depleting remedies, I think no one who continues to use them has any doubt.

The treatment proper for acute inflamation is the same now as formerly. Yet we do not bleed to subdue it neither so often nor so largely. It may not be necesseary, but I have often, very often, had my attention drawn to the subject by cases of phthisis following an attack of inflamation of the lungs; and I believe that if we were too active in our former treatment of disease, we are often now found at the other extreme.—*Transactions N. Y. State Society.*

